# Improving Environmental Efficiency of Reverse Filling Cementitious Materials through Packing Optimization and Fiber Incorporation

**DOI:** 10.3390/molecules26030647

**Published:** 2021-01-27

**Authors:** Yang Liu, Lou Chen, Keren Zheng, Qiang Yuan

**Affiliations:** School of Civil Engineering, Central South University, Changsha 410075, China; soyoung@csu.edu.cn (Y.L.); zhengkeren@csu.edu.cn (K.Z.); yuanqiang@csu.edu.cn (Q.Y.)

**Keywords:** reverse filling, packing density, optimization, environmental impact, cement use efficiency

## Abstract

To improve the environmental efficiency of the reverse filling system, three strategies aim to optimize the packing density, and the mechanical property were adopted in this study. Based on the compressive packing model (CPM), the relationship between the D50 ratio and maximum theoretical packing density for a reverse filling system with 25% and 30% superfine Portland cement was established. For comparison, silica fume and steel fiber were also added to the reverse filling system, respectively. The improvement of packing density by adjusting the D50 ratio was verified through the minimum water demand method, CPM, and modified Andreasen and Andersen (MAA) model. Compared to the reverse filling system added with 3 wt % silica fume, which possesses a comparable mechanical property with the optimized group (adjusted D50 ratio), the incorporation of steel fiber shows a more significant increase. The environmental efficiency of all the samples was quantified into five aspects through the calculation based on the mix proportion, compressive strength, and hydration degree. The comprehensive evaluation demonstrated that the optimized reverse filling system exerts a lower environmental impact and possesses a much higher cement use efficiency compared to the majority of ultra-high performance concrete (UHPC)/ ultra-high performance fiber-reinforced concrete (UHPFRC) reported in published papers.

## 1. Introduction

Due to its easy availability of raw materials, low-cost, ease of fabrication, and robustness, concrete is nowadays the most widely used construction material. Cement, one of the main components of concrete, accounts for the majority of embodied CO_2_ in concrete. According to statistics [1,2], the cement industry contributes 5–7% of anthropogenic CO_2_ emissions globally. It is estimated that global cement production is to increase between 12% to 23% by 2050, with the growing population and urbanization [3]. The tremendous quantity of cement used poses an increasingly urgent need to reduce the embodied CO_2_ for the cement and concrete sector worldwide.

The use of ultra-high performance concrete (UHPC) materials can noticeably lower the CO_2_ footprint in terms of the reduced size and prolonged service life as UHPC possesses both better mechanical property and durability compared to normal concrete. It has been reported that the UHPC solution can save about 73% of raw materials when building an *L*-shaped wall [4]. Technically, large amounts of cement, superfine powder, or even nanoparticles are required in order to form a highly dense packing structure in these high-performance cementitious materials [5,6]. For instance, Yu et al. [7] employed modified Andreasen and Andreasen particle-packing model to prepare UHPC, which consists of binder content around 655–920 kg/m^3^, fine quartz powder (D_50_ = 30 um), and limestone powder (D_50_ = 10 um). The compressive strength reached above 140 MPa at the age of 28 d. UHPC with compressive strength over 120 MPa (28 d) and noticeably reduced sorptivity, passed charge, as well as migration coefficient, were prepared by Arora et al. [8] via separation optimization of binder and aggregates gradation based on the compressive packing model. Despite the superior mechanical performance and improved durability; however, the hydration degree of cement in the typical UHPC system is only between 30% and 40% at the age of 28 d [9], which indicates a large quantity of anhydrate cement particles serves as an inert component with both high-cost and embodied CO_2_ [10,11,12,13].

**Figure 1:**
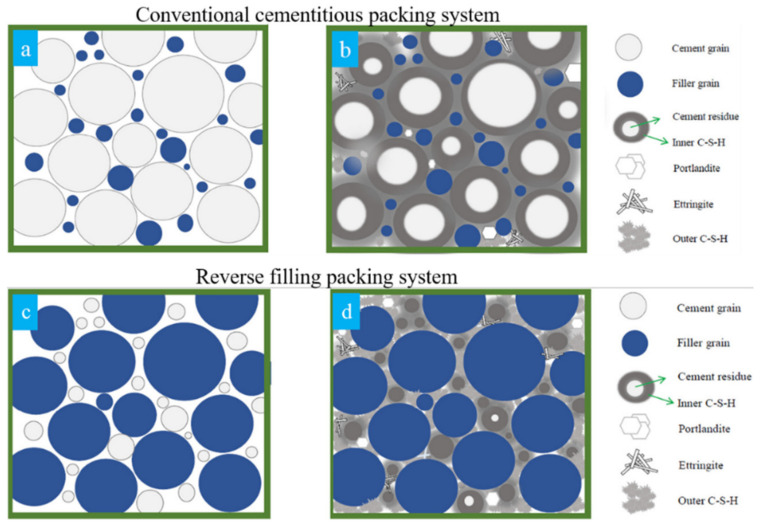
Schematically comparison of packing system between conventional cementitious system and reverse filling system (**a**,**c**) before and (**b**,**d**) after hydration, from [20].

To comprehensively understand the conception of the reverse filling system, a comparison between the conventional cementitious materials and the reverse filling system with respect to the packing system is presented. As demonstrated schematically in Figure 1a,b, large amounts of fine particles, especially silica fume, are commonly used to fill the space between the grain structure consists of Portland cement particles in the conventional packing system of UHPC, UHPFRC, or RPC. For instance, the content of cement and silica fume in UHPC is around 1100–1300 kg/m^3^ and 20–26 wt % in typical UHPFRC [14,15,16]. However, the limited available space suppressed the continuous hydration, resulting in a lowered hydration degree as well as the use of efficiency [17]. In comparison, in the reverse filling system, as shown in Figure 1c,d, instead of using cement as coarse particles to construct the skeleton of the packing system, the initial packing framework is built up by using inert coarse particles such as limestone and quartz. Superfine cement particles are then added to fill the space and bind these coarser particles during the hydration process. The substitution of Portland cement by inert filler in the main grain structure contributes to a larger reduction of cement content. In addition, the use of superfine cement could result in an enhanced hydration rate and degree due to the reduced size effect [18,19].

In our previous study, a binary reverse filling cementitious material was designed based on the compressive packing model (CPM) [20]. Superfine cement (mean size D_50_ = 4.0 μm) was used to fill the voids in the packing structure composed of larger limestone grains (mean size D_50_ = 14.4 μm) in order to attain high packing density and low cement use simultaneously. Results showed that the measured packing density could reach the maximum value (~0.752) when the superfine cement content was adjusted to 25 wt %, which is around two times lower than that in typical UHPFRC mix proportion [14,15,16]. Correspondingly, the compressive strength and hydration degree can reach 74.3 MPa and ~93% at 90 d, respectively, without adding fiber or silica fume. Meanwhile, in contrast with UHPC/UHPFRC with similar strength levels reported in published papers, a significant decrease in the mass fraction of unhydrated cement content and embodied CO_2_ (per kg binder) was also achieved.

Nevertheless, the environmental efficiency for reverse filling cementitious materials needs further evaluation and improvement because of the use of superfine cement, which consumes much higher energy for grinding than normal fineness Portland cement. In addition, microstructural experiments in the previous study also revealed that there is still room for improvement in terms of the packing density since visible voids were observed in its pore structure [20].

In this study, the environmental efficiency of reverse filling cementitious materials is improved through optimizing the packing density and incorporating steel fiber. The packing density was first improved by adjusting the size ratio of the limestone filler and superfine cement; then, silica fume was incorporated for further densification. Steel fiber was used to enhance mechanical properties, hence improving environmental efficiency. The improvement on environmental efficiency was thereafter evaluated using different indices, including cement use efficiency, binder index, clinker index, embodied energy, and CO_2_. This study provides a new sight into developing a high-performance cementitious system with low carbon emission and high-efficiency in cement use.

## 2. Materials and Methods

### 2.1. Raw Materials

**Figure 2:**
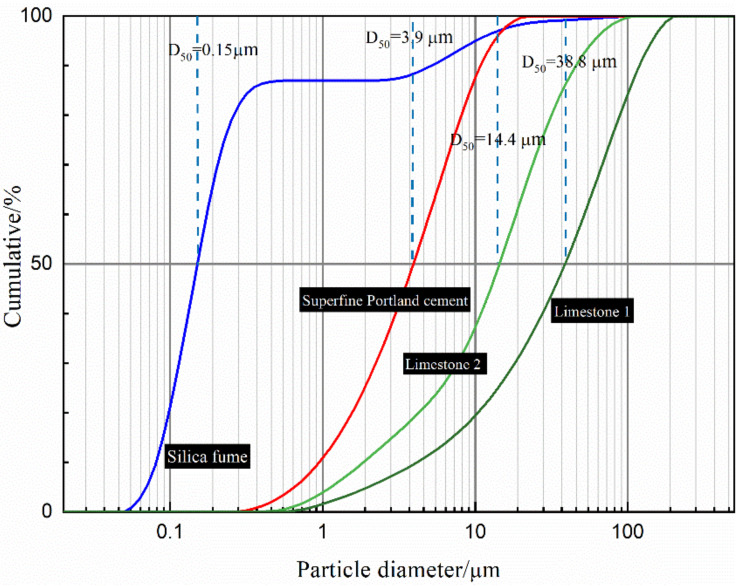
Particle size distribution curve of raw materials.

Superfine Portland cement (Type P·I, similar to CEM II, provided by Tangshan Polar Bear Building Materials Company, Tangshan, China), which conforms to according to GB/T 35161-2017, was used in this study. Silica fume and two limestone powders (with D_50_ of 14.4 μm and 38.8 μm, respectively) were used in this study. The particle size distribution, composition, and related physical properties of used raw materials are shown in Figure 2 and Table 1. The actual packing density of superfine cement and limestone were measured by the minimum water demand (MWD) method proposed by Laboratoire Central des Ponts et Chaussees (LCPC) [21]. A polycarboxylate-based superplasticizer (ViscoCrete^®^3301H, 55% solid content) from Sika China was used to reduce water demand and facilitate the dense packing. The length and diameter of steel fiber used in this study are 12 mm and 0.2 mm.

### 2.2. Mixing and Casting Procedure

**Figure 3:**
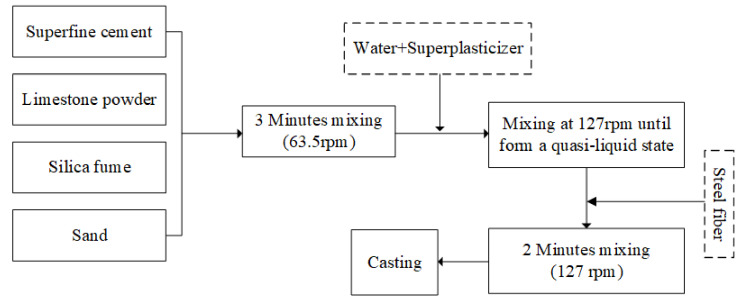
The mixing procedure to prepare reverse filling cementitious material.

A forced mixer with a rotating blade was employed to prepare samples. The detailed procedure is illustrated in Figure 3. First, the raw materials were added and mixed at dry-state with a slow rotating rate of 63.5 rpm for 3 min to improve the uniformity. Then water and superplasticizer were gradually added, and the spinning rate was turned up to 127 rpm to the powder turns into a quasi-liquid state, then the obtained mixture was mixed at 63.5 rpm for another 2 min. In the case of steel fiber incorporation, steel fiber was added into the homogeneous mixture at the mixing rate of 63.5 rpm.

Mortar and paste specimens of 160 × 40 × 40 mm^3^ were cast for mechanical property tests. The prism specimens were covered with plastic film and stored at 20 °C before demolding. After demolding at 1day, all the prisms were moved to a climate chamber (20 °C, ≥95% RH) until the scheduled ages. Paste samples for microstructure analysis were cast into airtight plastic vials (Φ 36 mm × 65 mm) and stored at 20 °C. After hardening, a few drops of deionized water were added to the plastic vials to keep the paste wetting.

### 2.3. Flowability

The flowability was measured utilizing the mini cone (Φ 60 × 36 mm^2^) test. During the test, the cone was filled with the mixture then lifted straight upwards to allow the mixture to flow freely. Once the flow stops, two diameters perpendicular to each other of the final spread were measured. The mean value was recorded as the flowability value for each mixture. The dosage of the superplasticizer was dependent on the flowability test with a target value of 230 ± 15 mm.

### 2.4. Mechanical Property

The compressive and flexural strength were measured according to Chinese standard GB/T 17671-1999 (similar to ASTM C109) at a loading rate of 2.4 kN/s and 50 N/s, respectively. The flexural strength test was first carried out on three prisms. Then six cube samples (40 × 40 × 40 mm^3^) obtained after the flexural test were subjected to the compressive strength test.

### 2.5. Hydration Degree

For the microstructural test, paste samples were cut into slices at the age of 90 d, then subjected to the hydration stoppage procedure recommended by Ruben Snelling [22]. X-ray diffraction (XRD) patterns of treated samples were collected using an X-Pert3 Powder (Panalytical. B. V, Netherland) diffractometer with CuKα radiation generated at 40 mA, and 40 kV. Samples were stepped scanned from 5° to 65° at 0.02° 2θ steps integrated at the rate of 2°/min. Quantitative phases analysis was performed using the Rietveld method with an external standard (TiO_2_, rutile phase) strategy, and phase contents were normalized to anhydrous mass (the ignited mass at 550 °C). Thermalgravimetric analysis (TGA) was carried out as a supplement to the XRD test. The powdered samples (~50 mg for each measurement) were tested from room temperature to 950 °C at a heating rate of 10 °C/min under an N_2_ atmosphere using a TGA 2(SF)(Mettler Toledo, Switzerland).

The content of chemical bound water was calculated from the mass loss below 550 °C based on the TGA measurement. The hydration degree was calculated according to Equation (1):

(1) $$DoH=1-\frac{w_{C_{3}S}(t)+w_{C_{2}S}(t)+w_{C_{3}A}(t)+w_{C_{4}AF}(t)}{\left(w_{C_{3}S}(t_{0})+w_{C_{2}S}(t_{0})+w_{C_{3}A}(t_{0})+w_{C_{4}AF}(t_{0}))\times (1-H_{2}O_{bound}\right)}$$

where w(t) and w(t_0_) stands for the remaining mass fraction at the age of t and unreacted state t_0_, H_2_O bound stands for the content of chemical bound water obtained from TGA.

### 2.6. Environmental Efficiency

A comprehensive assessment was carried out to evaluate the environmental efficiency of reverse filling cementitious materials. Five parameters, including the binder index, clinker index, embodied energy, embodied CO_2_, and content of unreacted cement, were calculated. The obtained results were also compared to that in typical UHPC from published papers [7,10,11,23]. It should be noted that the binder and clinker were referred to the number of cementitious materials and cement content that is required to produce one unit of strength (1 MPa) [24]. The binder index (bi) and clinker index (ci) were calculated according to the following equations:

(2) bi=b/σ

(3) ci=c/σ

where b and c are the total amounts of binder materials and cement per cubic meter (kg/m^3^). σ is the compressive strength at 28d. It should be noted that the limestone powder used in this study was not counted in the binder materials because it mainly works as inert particles. The hydration degree was also obtained from the XRD and TGA test at 28 d. The embodied energy and CO_2_ were calculated from the mix proportion. The data of embodied energy and CO_2_ for each constituent in both the reverse filling system and UHPC are summarized in Table 2.

## 3. Improvement Approaches

To improve the environmental efficiency of reverse filling cementitious materials, three approaches were used and compared to each other in this study. First, the packing was optimized by increasing the mean size of the limestone filler. Second, silica fume was used to further increase the initial packing density and reduce the porosity of the hardened materials through its pozzolanic reaction. Third, steel fiber was incorporated in order to enhance the mechanical properties of reverse filling cementitious materials, hence improving environmental efficiency.

Research conducted by De Larrard [29] implies that the theoretical maximum packing density for a binary packing system would increase with the decrease in the ratio between the size of fine and coarse particles (from 0.65 to 0.1).

In this study, D_50_, the diameter below which the cumulative portion accounts for 50%, is used to present the mean size of limestone filler or superfine cement, and the D_50_ ratio is referred to as the ratio between fine and large particles hereinafter in this study.

Hence, to obtain a lower value of the D_50_ ratio, a coarse limestone was incorporated. Second, since the previous study has revealed that there are still some small voids and a certain content of the portlandite phase in the binary reverse filling system, adding the supplementary cementitious materials (SCMs) with small particle size can be an ideal approach to address both the problem. Therefore, silica fume, which possesses a smaller particle size and relatively high pozzolanic reactivity, was blended in the reverse filling system. Third, steel fiber was incorporated in order to improve the mechanical property and ductility.

In this study, the compressive packing model (CPM) is utilized to optimize the packing density and design the mix proportion, which is shown as follows:

(4) $$\gamma_{i}=\frac{\beta_{i}}{1-\sum_{j=1}^{i-1}[1-\beta_{i}+b_{ij}\beta_{i}(1-1/\beta_{j})]y_{j}-\sum_{j=i+1}^{n}\left[1-\alpha_{ij}\beta_{i}/\beta_{j}\right]y_{j}}\gamma =\underset{1\leq i\leq n}{\min}\gamma_{i}$$

where d_i_ is the diameter of i grain class; γ and γ_i_ is the virtual packing density for the whole system and i grain class, respectively; y_i_ is the volume fraction for a particle with a diameter of d_i_; β_i_ is the residual packing density for a particle with a diameter of d_i_; α_ij_ is the loosening effect coefficient; b_ij_ is the wall effect coefficient. According to the research conducted by De Larrard [29], the relationship between the virtual packing density γ and the actual packing density Ø_c_ can be expressed as follows:

(5) $$K=\sum_{i=1}^{n}K_{i}=\sum_{i=1}^{n}\frac{y_{i}/\beta_{i}}{1/\phi_{c}-1/\gamma_{i}}$$

where K is the compaction index, depending on the process of building the packing structure. To calculate the actual packing density, particle size distribution (PSD), packing density(Ø) of the used superfine Portland cement (SPC), limestone powder (LP), and compaction index (K) were inputted. The value of K was set to be 6.7, according to [29]. The packing density for superfine portland cement and limestone powder were measured by the MWD as mentioned above.

**Figure 4:**
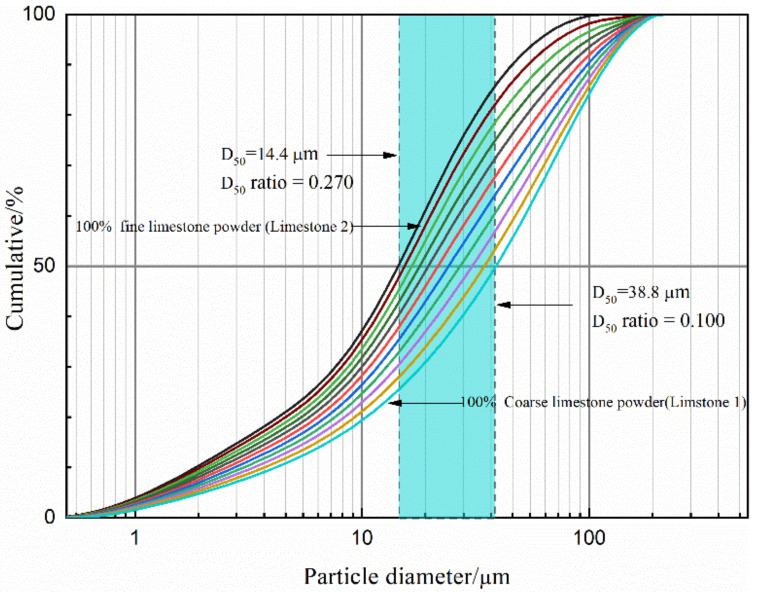
The particle size distribution of the mixed limestone powders of limestone 1 and limestone 2.

As can be seen from Figure 4, D_50_ of the mixed limestone powder increases from 14.1 μm to 39.9 μm with the increasing content of limestone 1 from 0% to 100%, thus mixed limestone powder with various D_50_ can be obtained. Correspondingly, the D_50_ ratio of superfine cement to mixed lime limestone powder varies between 0.270 (not 0.264) and 0.100 continuously, given a fixed D_50_ value (3.91 μm) of superfine cement.

**Figure 5:**
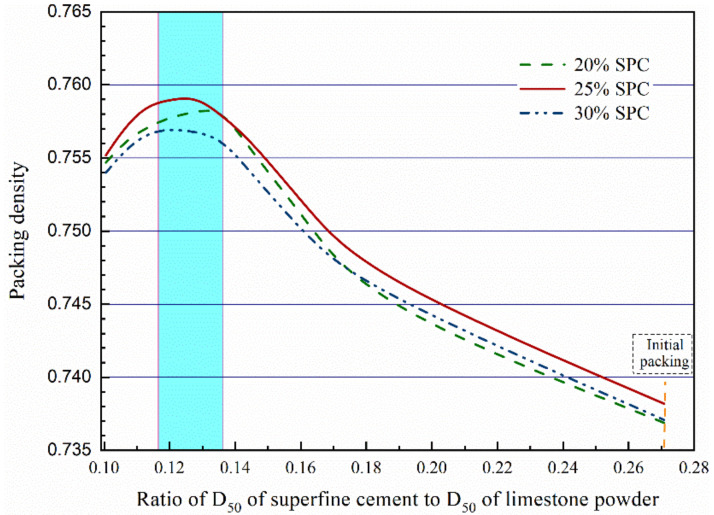
Effect of the D_50_ ratio between superfine portland cement and limestone powder on the packing density.

As shown in Figure 5, for the binary system with different content of superfine Portland cement (D_50_ = 3.9 μm), the estimated packing density calculated from the CPM model reaches the maximum value when the ratio of D_50_ locates between 0.116 and 0.136. This demonstrates that the increase of the D_50_ size of limestone powder can improve the packing density of the binary reverse filling system.

**Figure 6:**
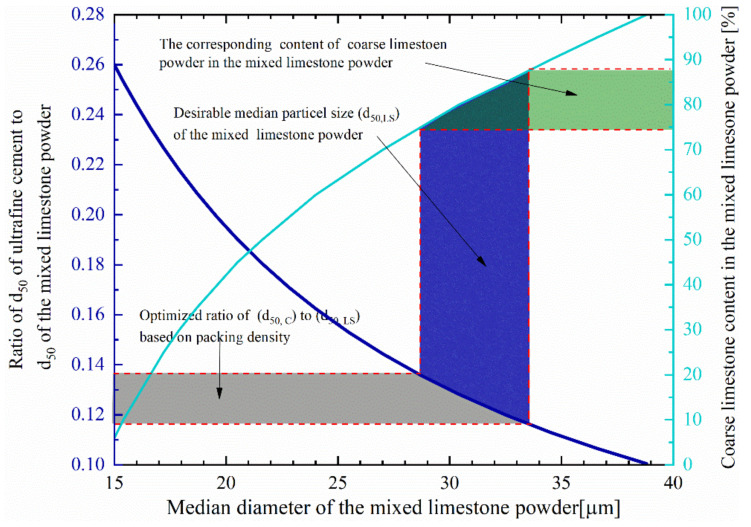
Relation between the size ratio, D_50_ of the mixed limestone powder, and the needed content of coarse limestone powder.

Figure 6 presents the correlation between the size ratio, D_50_ value, and the content of coarse limestone powder (limestone 1). To obtain the desired size ratio (0.116–0.136), which corresponds to the highest packing density, the D_50_ of mixed limestone powder and content of limestone 1 should locate between 28.7 and 33.6 μm and 74−88%. Therefore, in this study, the content of limestone 1 was chosen to be 80%. Correspondingly, the D_50_ and size ratio is 30.4 μm and 0.128, respectively.

The incorporation of mixed limestone powder renders the binary system to achieve a lower *w*/*b* ratio. The optimized binary systems with a lowered water to powder ratio (*w*/*p*) of 0.11 and water to cement ratio (*w*/*c*) of 0.44 and 0.37 were formulated, as shown in Table 2. In addition, the observation from the previous study indicates small voids still exist in the microstructure [20]. In typical mix proportion of UHPC, silica fume, which possesses fine particle size and high pozzolanic reactivity, was normally incorporated to further increase the packing density and produce the secondary C–S–H at the expense of portlandite phase [5,11]. The dosage of silica fume in typical UHPC varies from 20%−35% of cement content and decreases with the *w*/*c* ratio [5]. However, owing to its very fine size, high specific surface area, and content of carbon, the addition of silica fume decreases the fluidity. In this study, 3 wt % silica fume was added into the binary reverse filling system as the flowability for all samples was controlled within 230 ± 15 mm in order to avoid the effect of variable fluidity on the packing density. What is more, the incorporation of steel fiber has been reported to improve both the compressive and tensile strength [7,30]; the improvement of the mechanical property may further reduce the environmental impacts, meanwhile, in order to evaluate and compare the environmental impact with typical UHPC and UHPFRC. Mortar samples and fiber-reinforced samples were also prepared in this study. Considering the adverse effect of adding steel fiber on the flowability, the dosage of it was fixed at 2% (volume fraction). The mass ratio between all the powders and sand was set to be 2.5. The mixed proportion of all the samples are shown in Table 3.

## 4. Results and Discussion

### 4.1. Mechanical Property

#### 4.1.1. Effect of Optimized Packing Density and Cement Proportion

**Figure 7:**
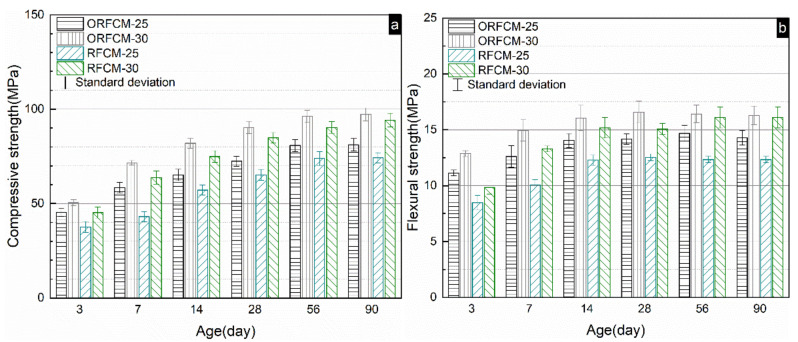
Compressive (**a**) and flexural (**b**) strength of optimized and non-optimized limestone-ultrafine Portland cement reverse filling cementitious system.

The compressive and flexural strength for optimized reverse filling cementitious materials from 3 days to 90 days are presented in Figure 7a,b). As shown, both the compressive and flexural strength increased with the content of superfine Portland cement. Sample ORFCM-30 exhibited both the highest compressive and flexural strength regardless of age. This is ascribed to the reduced water to cement (*w*/*c*) ratio. Although the water to powder (*w*/*p*) ratio for optimized reverse filling materials is fixed at 0.12, the actual *w*/*c* ratio for ORFCM-25 and ORFCM-30 is 0.48, and 0.4, respectively. According to research conducted by Powers [31], the minimum required *w*/*c* ratio for fully hydrated Portland cement is around 0.38. Theoretically, a *w*/*c* ratio higher than 0.38 would give rise to more voids, which coarse the microstructure and weaken the mechanical property. It is obvious that the non-optimized samples also exhibit a similar strength development, i.e., gradually increased to its maximum value at around 56d and stabilized afterward. It should be noted that the compressive strength of all the samples at 1d was not presented because of the retarding effect brought by the addition of large amounts of superplasticizer. The comparison of mechanical property between the optimized and non-optimized group are shown in Figure 7. For sample ORFCM-25, the compressive and flexural strength reached 81.5 MPa and 14.8 MPa at 90 d, which is around 10% higher than that of RFCM-25. As for sample ORFCM-30 and RFCM-30, the gap between their mechanical property gradually diminished with the increasing curing time. At the age of 90 d, both the compressive and flexural strength increased to about the same level.

#### 4.1.2. Effect of Silica Fume and Steel Fiber

**Figure 8:**
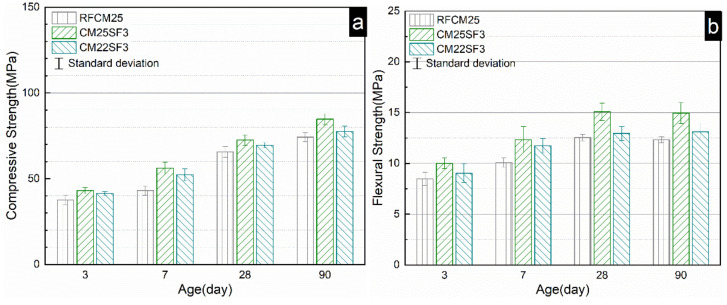

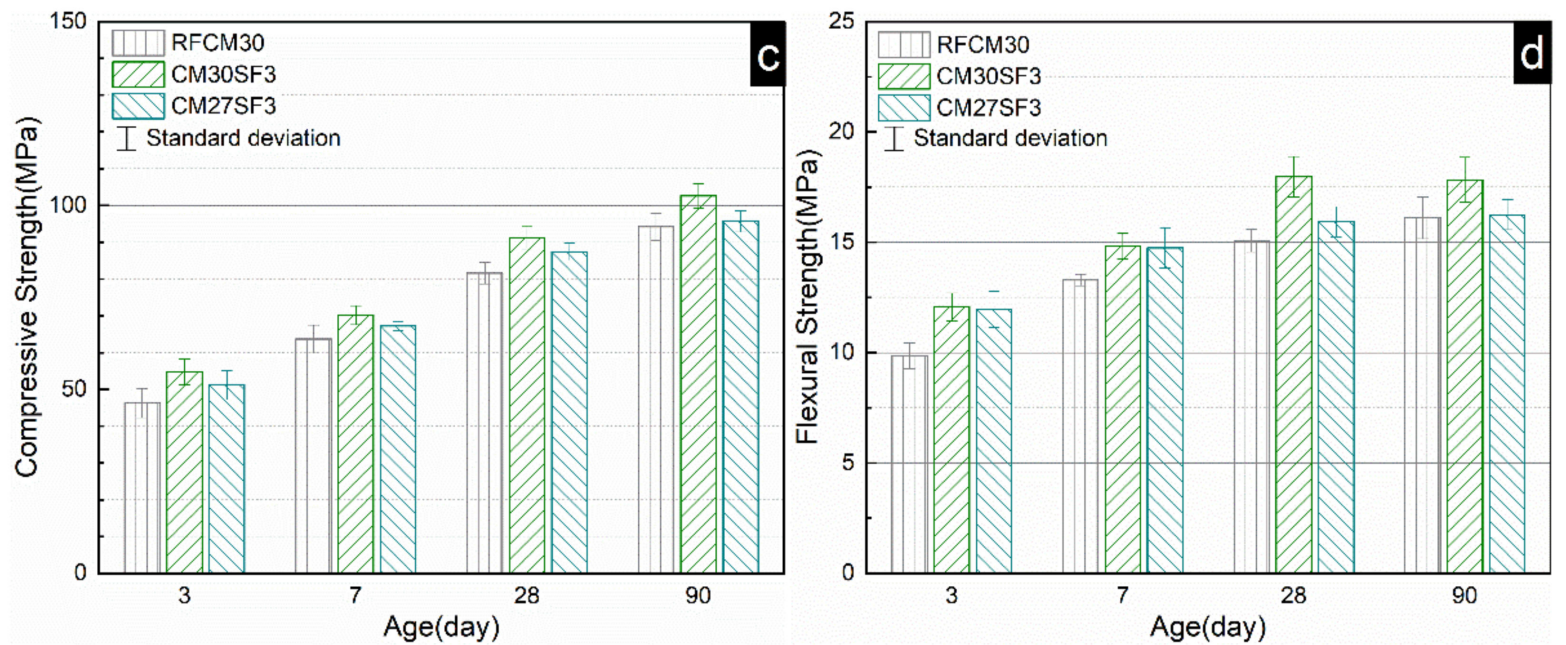
Compressive strength (**a**,**c**) and flexural strength (**b**,**d**) of limestone powder-superfine Portland cement–silica fume system.

The mechanical property of the reverse filling system blended with silica fume and steel fiber is shown in Figure 8. As can be seen, regardless of the cement content, the incorporation of 3% silica fume generally enhanced the mechanical property to a different extent compared to RFCM25 and RFCM30. The enhancement in mechanical property is more significant for RFCM25. For instance, the compressive and flexural strength of RFCM25 was increased by ~13% and ~25% at the age of 90d, respectively. The very low particle size of silica fume (D_50_ = 0.15 μm) allows it to fill the voids in the matrix, further increasing the packing density. What is more, the intrinsic amorphous SiO_2_ content in silica fume can consume portlandite to form extra C–S–H gel to densify the microstructure. It is clear that the substitution of limestone powder by silica fume in both the RFCM25 and RFCM30 systems produced a more remarkable enhancement in the mechanical property than that in substitution of superfine Portland cement. The compressive of CM25SF3 and CM30SF3 was improved by around 13% and 8.5% compared to RFCM25 and RFCM30 at 90 d, reaching 84.7 MPa and 102.6 MPa. Correspondingly, their flexural strength was also elevated by around 20% and 18.6%, attaining 15.2 MPa and 17.8 MPa at 90 d, respectively.

**Figure 9:**
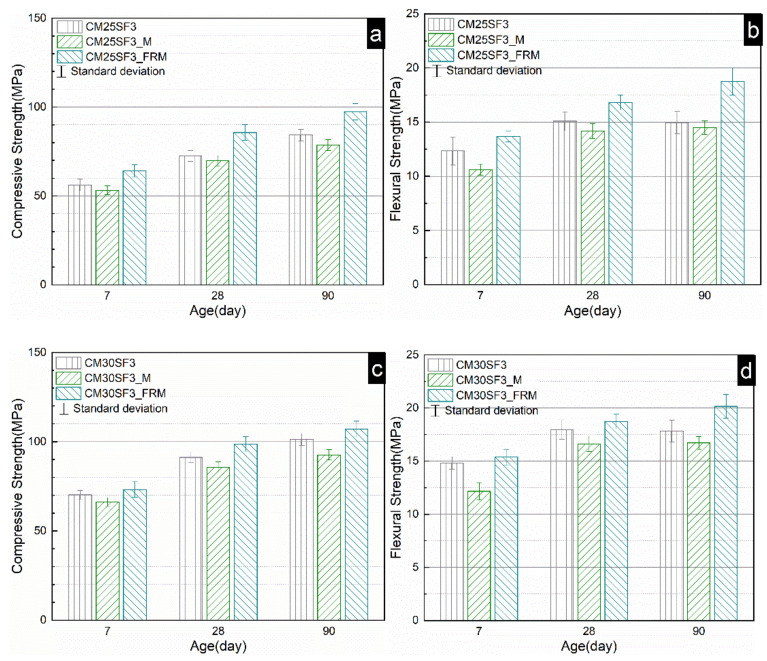
Compressive strength (**a**,**c**) and flexural strength (**b**,**d**) of paste, mortar, and fiber-reinforced reverse filling system. (*p* = paste, M = mortar, FRM = fiber reinforcement mortar).

Since the CM25SF3 and CM30SF3 displayed a better mechanical property, their mechanical property of mortar and fiber-reinforced mortar were further investigated and compared. As shown in Figure 9, the addition of steel fiber noticeably improved both the compressive and flexural strength. The compressive strength of CM30SF3_FRM and CM25SF3_FRM ascended to 108.3 MPa and 97.4 MPa while the flexural strength raised to 20.2 MPa and 18.7 MPa, respectively.

### 4.2. Hydration Degree

**Figure 10:**
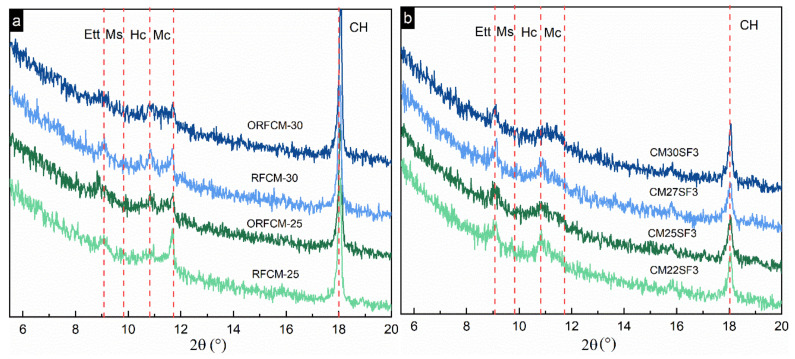
XRD patterns of opitimized and non-optimized samples (**a**) and samples (**b**) blended with silica fume at the age of 90 d (Ett = ettringite, Ms = monosulfate, Hc = hemicarbonate, Mc = monocarbonate).

Figure 10 shows the amplified regional XRD patterns of all the binary and ternary reverse filling system at 90 d. As shown, obvious peaks of mono and/or hemi-carbonate were observed instead of monosulfate. This is due to the presence of calcite from limestone powder, which reacts with the Al-bearing phase or substitutes the SO_4_^2−^ by CO_3_^2−^ in monosulfate. The incorporation of silica fume significantly reduced the content of portlandite mirrored by the lowered intensity of CH peak. Compared to the non-optimized binary reverse filling system, the intensity of the monocarbonate peak is lower. This is because of the enlarged size of limestone powder, which affects its reactivity with Al-bearing phases and reaction rate of hydration [32]. In a reverse filling system blended with silica fume, monocarbonate was not detected, which indicates the addition of silica fume affected the reaction between calcite and Al-bearing phase.

**Figure 11:**
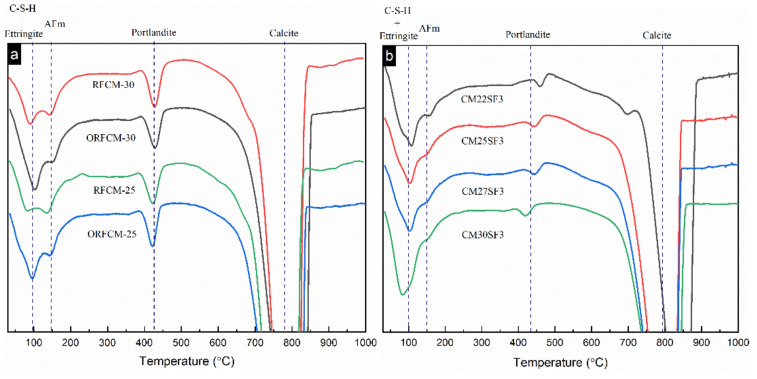
DTG curves of optimized and non-optimized samples (**a**) and samples blended with silica fume (**b**) samples at 90 d.

The DTG curves of all the paste samples are shown in Figure 11. Generally, four peaks corresponding to C–S–H and ettringite, AFm, portlandite, and calcite can be observed in all curves. It is remarkable that the peak of portlandite, which locates between 400 to 450 °C in a reverse filling system blended with silica fume, was much lower than that in the binary reverse filling system. This is also consistent with the trend implied by XRD patterns (Figure 10). Moreover, the peak of C–S–H and ettringite in reverse filling system incorporated with silica fume is more pronounced than that in the rest of other blended systems.

**Figure 12:**
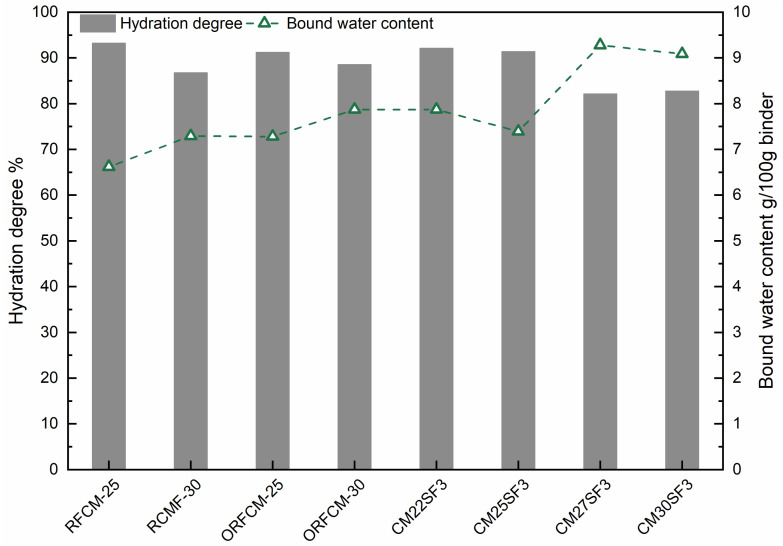
Hydration degree and bound water content of all the samples at 90 d.

Figure 12 displays the hydration degree of cement and the content of chemical bound water normalized to 100 g binder. As can be seen, despite sample CM27SF3 and CM30SF3, the hydration degree of all the other samples reached around or above 90% at 90 d. The content of chemical bound water seems to be inconsistent with the hydration degree for sample CM27SF3 and CM30SF3. The possible reason for this is due to the addition of silica fume, which leads to a denser packing structure compared to RFCM-30 and ORFCM-30. Consequently, the available space for subsequent hydration in paste samples blended with silica fume is less than that in RFCM-30 and ORFCM-30 [33]. In addition, the silica fume reacts with the portlandite to form secondary C–S–H, contributing to an increase in the content of chemical bound water.

### 4.3. Packing Density and Its Correlation with Mechanical Property

**Figure 13:**
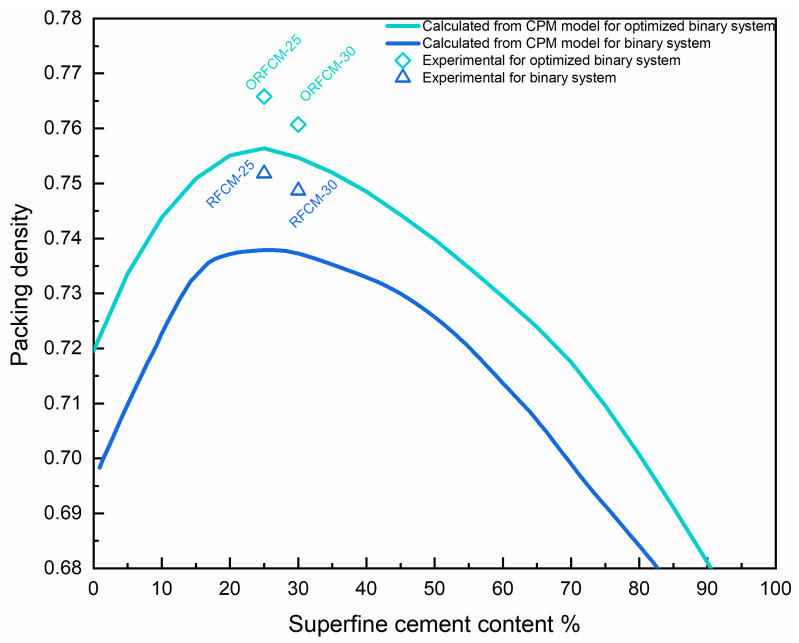
Packing density was calculated from the compressive packing model (CPM) model and MWD) test.

Figure 13 shows the predicted and actual measured packing density for the limestone-superfine cement system and the optimized binary system. As can be seen, both the calculated and measured packing density for optimized binary are higher than those in the non-optimized system. The curve of calculated packing density exhibits a similar trend, i.e., reaching the maximum value when the content of superfine cement is around 20–30%.

The packing density of the optimized binary system can attain as high as ~0.766, which is improved by 23% compared to the highest value in the non-optimized binary system (RFCM25). In addition, it is clear that the value of measured packing density from the MWD method for both the optimized and non-optimized binary system is higher than the calculated value from the CPM model. The possible reason for this phenomenon is due to the addition of a polycarboxylic-based superplasticizer, which increases the water film thickness around the fine particles. Hence, the solid particles have a better dispersion in the matrix, resulting in an improved packing density [34]. In addition, the effect of superplasticizer was not accounted for in the compressive packing model.

Different from the discrete CPM model, which utilizes the mono-size particle packing to predict the packing density, the modified Andreasen and Andersen (MAA) model which belongs to the continuous model, was also widely used to design the mix proportion of UHPC [5]. In the MAA model, when the PSD curve of the powders satisfies Equation (6), the packing density of the system can achieve the maximum value.

(6) $$P(D)=\frac{D^{q}-D_{\min}^{q}}{D_{\max}^{q}-D_{\min}^{q}}$$

where D is the particle size, D_max_ and D_min_ are the maximum and minimum particle size, *q* is the distribution modulus. The value of distribution modulus *q* has a great influence on the ideal particle distribution curve. It has been reported that when the packing system is dominated by the fine particle, the value of q should be less than 0.25. Based on the work conducted by [7,35], the value of q is fixed at 0.23 in this study. The deviation between the ideal curve and the PSD curve for the blended system is calculated based on the residual sum of squares (RSS) as follows:

(7) $$RSS={\sum_{i=1}^{n}\left(P_{mix}(D_{i}^{i+1})-P_{ideal}(D_{i}^{i+1})\right)}^{2}$$

**Figure 14:**
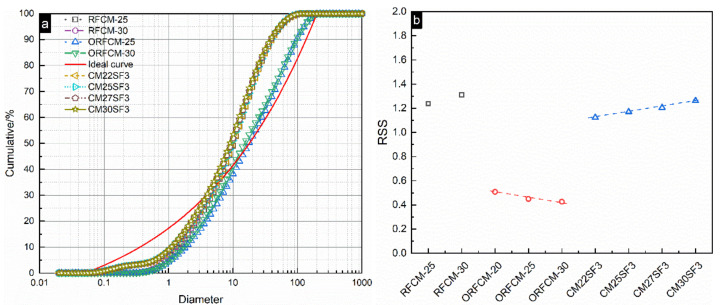
Particle size distribution (PSD) of reverse filling system and ideal system (**a**) and the residual sum of squares (RSS) value (**b**).

P_mix_ and P_ideal_ are the fractions of particles smaller than the size of D_i_ in the blended system and ideal PSD curve calculated from equation (6). Theoretically, the lower the RSS is, the closer to the ideal curve, the PSD curve of the blended system is. As can be seen from Figure 14, the PSD curve for the optimized binary system, i.e., ORFCM-25 and ORFCM-30, are closer to the ideal curve compared to all the other samples. The results of RSS are also consistent with the PSD curve. It is obvious that the incorporation of 3% silica fume can only slightly lower the RSS value between the reverse filling system and the ideal curve.

**Figure 15:**
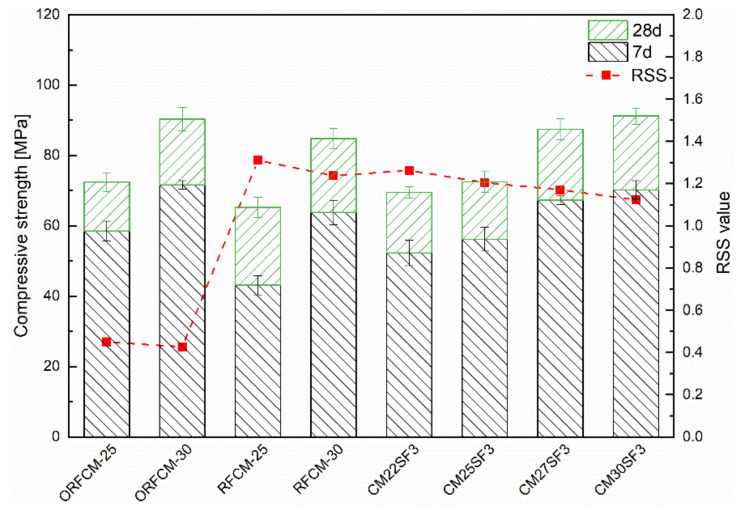
Relationship between compressive strength and packing density.

To reveal the effect of packing density on the mechanical property, the value of RSS and packing density calculated from the MAA and CPM model were plotted against the compressive strength at 7 and 28 d. As shown in Figure 15, the packing density indicated by the RSS value demonstrated that the higher content of superfine cement attributes to higher packing density. Correspondingly, samples consist of 30 wt % superfine cement exhibits a higher compressive strength at 7 d. In addition, the optimization of the packing system by adjusting the D_50_ ratio of mixed limestone powder exerts a remarkable improvement on both early-age compressive strength. The substitution of superfine cement by silica fume produced a less significant improvement on both mechanical property and packing density compared to that by adding 3 wt % silica fume. Moreover, although sample CM25SF3 and CM30SF3 possess a lower packing density, their compressive strength reaches a similar level with the optimized samples, namely ORFCM-25 and ORFCM-30. The possible explanation is due to the pozzolanic reaction of silica fume. Compared to the space-filling ability of silica fume at the initial packing state, the reaction between silica fume and portlandite resulted in secondary C–S–H, thus leading to a refined microstructure. Hence, the addition of silica fume may not manifest into an obvious increase of packing density, but the pozzolanic reaction leads to a densification of microstructure at a late-age.

### 4.4. Cement Use Efficiency and Environmental Impact

As already shown in Table 3, the content of silica fume and superfine cement used in the mortar sample of the reverse filling system were below 400 kg/m^3^ and 50 kg/m^3,^ respectively. The environmental impact of the reverse filling system was mainly derived from the superfine cement compared to that of all the other powders. Five ecological indices, including binder index, clinker index, the portion of unreacted cement, embodied CO_2_, and energy were calculated based on the mix proportion of 1 m^3^ (Table 3).

**Figure 16:**
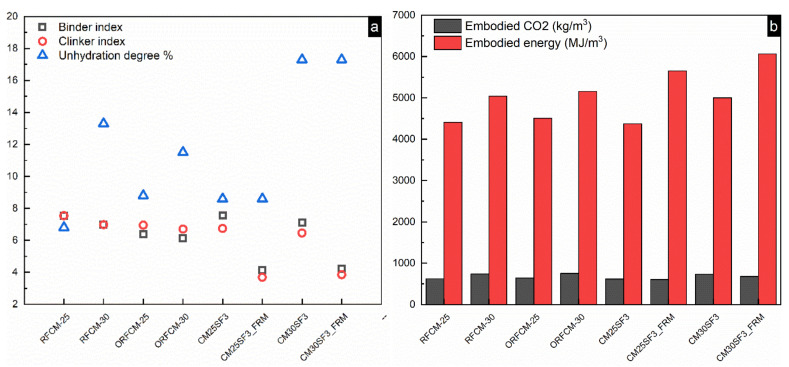
Comparison of environmental impact including unhydration degree, binder, and clinker index (**a**) and embodied energy and CO_2_ (**b**) within reverse filling system.

Figure 16 presents the value of five ecological indices for sample blended with 25% and 30% superfine Portland cement. As can be seen, the optimized group (ORFCM-25 and ORFCM-30) shows a marginal decrease of both binder and clinker index compared to the non-optimized group (RFCM-25 and RFCM-30). There is almost no difference between their value of embodied CO_2_ and energy. As for paste sample CM25SF3 and CM30SF3, although the addition of 3% silica fume reduces the embodied CO_2_ and energy, it led to an obvious increase of binder and clinker index as well as the portion of unreacted cement. Among all the samples presented in Figure 16, the incorporation of steel fiber significantly decreased the binder and clinker index since it improved the mechanical property, as already shown in Figure 9. Nonetheless, samples blended with steel fiber possesses the highest value of embodied CO_2_ and energy. This was ascribed to the higher environmental impact brought by steel fiber, as already implicated in Table 2.

**Figure 17:**
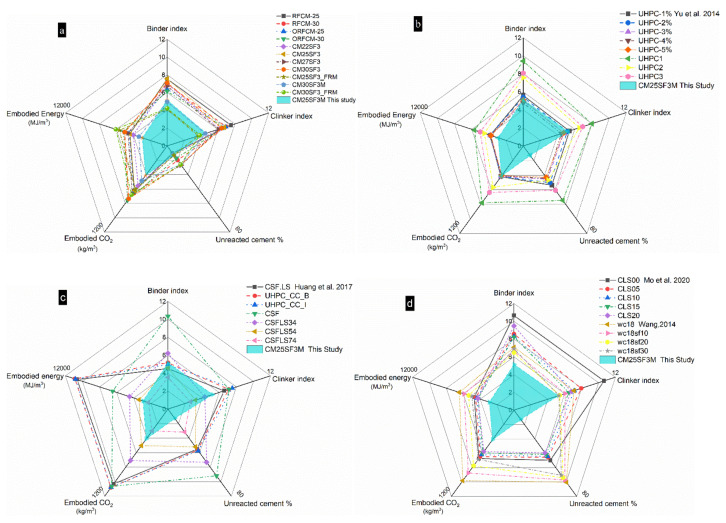
Comprehensive evaluation and comparison of the environmental impact between reverse filling system (**a**) and UHPC/UHFRPC (**b**–**d**).

To carry out a comprehensive evaluation of environmental impact, radar graphs which involve all the five ecological indices were plotted in Figure 17. As can be seen in Figure 17a, sample CM25SF3M exhibited better performance than all the other reverse filling systems as it occupies the smallest area among all the samples. Meanwhile, it also possessed a comparable value of both binder index and unreacted cement content in comparison with the optimized samples. Therefore, CM25SF3M was set as the reference sample for the reverse filling system and compared to the typical UHPC from published paper as hydration degree and strength at 28 d were studied [7,10,11,23,36,37]. The comparison in the five aspects between CM25SF3M and UHPC is shown in Figure 17b–d. It is clear that the values of the five indices in CM25SF3M were much lower than that in the majority of UHPC. For instance, compared to UHPC1 from [7] (shown in Figure 17b), there were around 47.8%, 25.3%, 50.4%, 48.7%, and 78.6% reduction corresponded to binder index, clinker index, embodied energy, embodied CO_2_, and the content of unreacted cement in CM25SF3M. Even compared to the optimized UHPC system [11], as shown in Figure 17c, the CM25SF3M still displayed a comparable performance. There are several reasons for this result. First, even though the superfine cement has higher embodied energy and CO_2_ compared to that of ordinary Portland cement, the content of ordinary Portland cement still accounts for a higher portion in the mix proportion compared to that in the reverse filling system (reduced to 25%). In addition, the mechanical property of the reverse filling system can reach a comparable level; meanwhile, the embodied energy and CO_2_ are remarkably reduced, which results in a decreased binder and clinker index. What is more, as presented in Table 3, owning to the reduced cement content, the *w*/*c* ratios for the reverse filling system are higher or around the theocratical *w*/*c* ratio for full hydration of cement [31]. Moreover, the superfine cement particles which fill into the voids of the packing structure of coarse limestone powder possess more space for hydration compares to that in the UHPC packing structure, where the relatively coarser cement particles are surrounded by finer particles. Hence, the abovementioned reasons attribute to better performance compared to typical UHPC/UHPFRC in the evaluation of environmental impact.

## 5. Conclusions

This study proposed and compared three approaches to further improve the packing density as well as environmental efficiency in the reverse filling system. The mechanical property and its correlation with packing density were studied and clarified. The environmental impacts of all reverse filling system were quantified in five aspects and compared to the typical UHPC system. The experiments and theoretical analysis lead to the following conclusions:

(1) By adjusting the D_50_ ratio and silica fume in the reverse filling system, the packing density can be further improved, as reflected from both the CPM and MAA model. These two strategies produce a comparable improvement in mechanical properties;

(2) The incorporation of steel fiber leads to a more remarkable increase in both compressive and flexural strength than the other two strategies;

(3) In terms of the environmental impact of the proposed three strategies, the optimization of the D_50_ ratio and incorporation of silica fume exert marginal improvement compared to the non-optimized sample. The addition of steel fiber significantly decreases the binder and clinker index while raises the embodied energy and CO_2_;

(4) The synthetic evaluation from five ecological aspects demonstrate that the reverse filling system has a much lower environmental impact compared to the majority of UHPC/UHPFRC. Especially, the cement use efficiency of the reverse filling system indicated by the hydration degree exhibits a pronounced improvement.

## Figures and Tables

**Table 1:** Chemical composition (wt %) and other properties of experimental materials.

		SiO_2_	Al_2_O_3_	Fe_2_O_3_	CaO	MgO	SO_3_	P_2_O_5_	Others + LOI	D_50_ (μm)	Density (g/cm^3^)
Cement		19.9	5.1	2.8	59.5	2.6	3.0	0.0	6.8	3.9	2.96
Limestone 1	1000 mesh	0.07	0.05	0.04	55.41	0.40	0.12	-	44.0	14.4	2.71
Limestone 2	100 mesh									38.8	2.73
Silica fume		95.2	0.30	0.07	0.28	1.68	1.01	0.32	1.14	0.15	2.21
		C_3_S	C_2_S	C_3_A	C_4_AF	Calcite	Gypsum	Bassanite	Quartz	Lime	actual packing * density ∅
Cement		58.8	14.0	6.6	8.6	6.6	4.3	6.6	-	0.4	0.656
Limestone 1	1000 mesh	-	-	-	97.4	-	-	-	2.6	-	0.696
Limestone 2	100 mesh										0.705

* measured from MWD method.

**Table 2:** Embodied CO_2_ and energy for raw materials used in this study.

Material	Embodied CO_2_ (kg/kg)	Embodied Energy (MJ/kg)	Reference
Ordinary Portland cement	0.83	4.6	[2]
Superfine Portland cement	1.15	6.6	[25]
Limestone powder	0.017	0.3	[2]
Calcined clay (metakaolin)	0.33	1.44	[26]
Silica fume	0	0.036	[27]
Quartz sand	0.02	0.85	[2]
Normal sand	0.005	0.1	[2]
Steel fiber	1.50	20.56	[27]
Water	0	0.2	[2]
Polycarboxylic-based superplasticizer	0.6	11.47	[28]

**Table 3:** Mix proportion of designed reverse filling paste and mortar.

Sample Code	Ultrafine Portland Cement	Limestone 1	Limestone 2	Silica Fume	Sand	Steel Fiber	Water	SP	*w*/*c*	*w*/*p*
	(kg/m^3^)									
RFCM-25	491.4	0.0	1474.2	0.0	0.0	0.0	235.9	59.0	0.48	0.12
RFCM-30	591.5	0.0	1380.2	0.0	0.0	0.0	236.6	59.2	0.40	0.12
ORFCM-25	502.9	1207.0	301.7	0.0	0.0	0.0	221.3	60.3	0.44	0.11
ORFCM-30	605.2	1129.8	282.4	0.0	0.0	0.0	221.9	60.5	0.37	0.11
CM22SF3	429.5	0.0	1464.3	58.6	0.0	0.0	234.3	58.6	0.55	0.12
CM25SF3	489.0	0.0	1408.3	58.7	0.0	0.0	234.7	58.7	0.48	0.12
CM27SF3	528.8	0.0	1370.9	58.8	0.0	0.0	235.0	58.8	0.44	0.12
CM30SF3	588.6	0.0	1314.5	58.9	0.0	0.0	235.4	58.9	0.40	0.12
CM25SF3M	321.4	0.0	925.6	38.6	514.2	0.0	154.3	38.6	0.48	0.12
CM25SF3_FRM	315.8	0.0	909.5	37.9	505.3	135.2	151.6	37.9	0.48	0.12
CM30SF3M	386.4	0.0	863.0	38.6	515.2	0.0	154.6	38.6	0.40	0.12
CM30SF3_FRM	379.7	0.0	848.0	38.0	506.3	135.4	151.9	38.0	0.40	0.12

## Data Availability

The data presented in this study are available on request from the corresponding author.
